# Hypogammaglobulinemia Causing Multiple Abscesses and Osteomyelitis of Calcaneus Following a Heel Puncture in a Preterm Neonate

**DOI:** 10.7759/cureus.36992

**Published:** 2023-04-01

**Authors:** Kanika Deora, Luna Khanal

**Affiliations:** 1 Pediatrics and Neonatology, Children's Hospital of Michigan, Detroit, USA; 2 Pediatrics, Children's Hospital of Michigan, Detroit, USA

**Keywords:** abscess, preterm, puncture wound, immunoglobulins, osteomyelitis

## Abstract

Osteomyelitis is a challenging diagnosis for a neonate. It might result from hematogenous spread or direct extension of a skin infection. The most common organism is *Staphylococcus aureus*. Preterm infants are at high risk for osteomyelitis because of their immature immune system, hypogammaglobulinemia, frequent blood draws, and invasive monitoring and procedures. We report a case of a male neonate born at 29 weeks by cesarean section, requiring intubation and transfer to the neonatal intensive care unit (NICU). At 34 weeks, he was noted to have an abscess in the lateral aspect of the left foot, which required incision, drainage, and antibiotic coverage of cefazolin as *S. aureus* was sensitive to penicillin. Four days later (34 + 4 weeks), a left inguinal abscess was noticed, which grew *Enterococcus faecium *on drainage, which is considered a contaminant initially, but one week later, another left-sided inguinal abscess was noticed growing *E. faecium*, and the patient was subsequently treated with linezolid.

Immunoglobulin levels were found to be low for IgG and IgA. After two weeks of antibiotics, a repeat x-ray of the foot demonstrated changes likely due to osteomyelitis. The patient received seven weeks of antibiotics targeting methicillin-sensitive staphylococcus and three weeks of treatment with linezolid for an inguinal abscess. A repeat x-ray of the lower left extremity after the patient received one month of antibiotics in an outpatient setting showed no signs of acute osteomyelitis in the calcaneus. Immunoglobulin levels continued to be low during the outpatient immunology follow-up. During the third trimester of pregnancy, the transfer of maternal IgG across the placenta begins, leading to lower IgG levels in premature infants and predisposing them to severe infections. The usual site of osteomyelitis is the metaphyseal region of the long bones, but any bone can be affected. The depth of penetration of the puncture site during routine puncture of the heel can cause a local infection. Early x-rays can aid in diagnosis. Antimicrobial treatment is frequently administered intravenously for two to three weeks and then switched to oral medication.

## Introduction

Osteomyelitis is a challenging diagnosis. The mode of infection can be hematogenous spread or direct extension of a skin infection after a latent period. The predisposing factors can be prematurity, skin or umbilical sepsis, or systemic infections. The most common bacterial pathogen causing osteomyelitis in children is *Staphylococcus aureus* in all age groups. The diagnosis can be clinically supported by blood culture or by pus aspirate found radiologically. Preterm infants are at high risk for osteomyelitis because of their immature immune system, frequent blood draws, invasive monitoring and procedures, and intravenous drug administration [1,2]. Immunoglobulin levels are physiologically lower in the first months of life, thus leading to a higher risk for recurrent infections in preterm infants [3,4]. We report a case of multiple abscesses of the inguinal region caused by *Enterococcus* and osteomyelitis of calcaneus caused by methicillin-sensitive *S. aureus* likely due to heel puncture from blood sampling in a preterm infant with hypogammaglobulinemia.

## Case presentation

The 29-week male baby in this case study was born by cesarean section with no spontaneous cry and required intubation at birth. His mother had severe pre-eclampsia and severe fetal heart tone deceleration. The preterm neonate was admitted to the neonatal intensive care unit (NICU) and required noninvasive respiratory support as well as parenteral and enteral nutrition. Doctors found that he had a moderate atrial septal defect, anemia, osteopenia of prematurity, hyperbilirubinemia of prematurity, and bronchopulmonary dysplasia.

At one month old, around 34 weeks, the baby was noted to have an abscess in the lateral aspect of the left foot (1.5 cm x 1 cm), fluctuant in nature. Initially, the x-ray was compromised due to his prematurity. However, the ultrasound demonstrated soft tissue cellulitis with an abscess. Incision and drainage were performed, and the patient was started empirically on ceftazidime and vancomycin, which was later switched to cefazolin as it grew *S. aureus* sensitive to penicillin. Four days after (34 + 4 weeks), a left inguinal abscess was noticed, which on drainage grew *Enterococcus* species which was considered as a contaminant initially, but one week after, another left-sided inguinal abscess was noticed, which grew *Enterococcus faecium* again sensitive to linezolid, and treatment was started. The initial newborn screening of the patient demonstrated suspected severe combined immunodeficiency, possibly due to low levels of T-cell receptor excision, but the repeat was negative. The infant was evaluated for possible immunodeficiency disorders, and the lab work showed lower levels of immunoglobulins. IgG was initially at a level of 145, IgA was undetectable, and IgM was at a level of 25. Two weeks later, repeat IgG was lower, dropping from 145 to 94. IgA was less than <10, which was still low.

**Figure 1:**
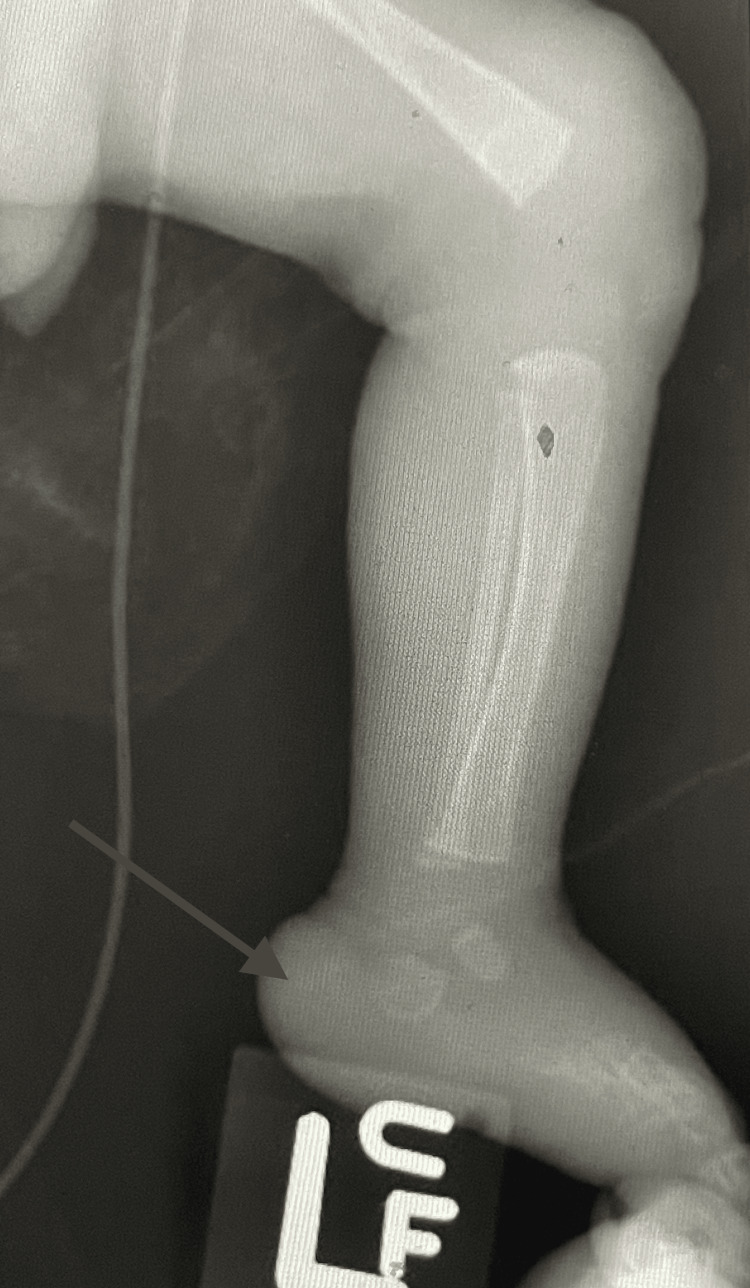
Arrow showing cortical irregularity suggesting osteomyelitis of calcaneus

Doctors performed a repeat x-ray of the foot after two weeks of antibiotics, which showed slight lucency at the posterior aspect of the calcaneus, with cortical disruption representing changes from osteomyelitis (Figure 1). MRI of the left foot was recommended. However, this was unable to be obtained as there is no MRI protocol for an infant of the patient's size. Blood culture showed no growth, while C-reactive protein was elevated to 79.8 with a white blood cell count (WBC) of 24.4, with no other systemic signs of infection. The changes seen in the x-ray of the left foot (Figure 1) prompted presumed osteomyelitis of the left calcaneus, and the patient received seven weeks of antibiotics targeting methicillin-sensitive staphylococcus and seven weeks of treatment of linezolid for inguinal abscess.

**Figure 2:**
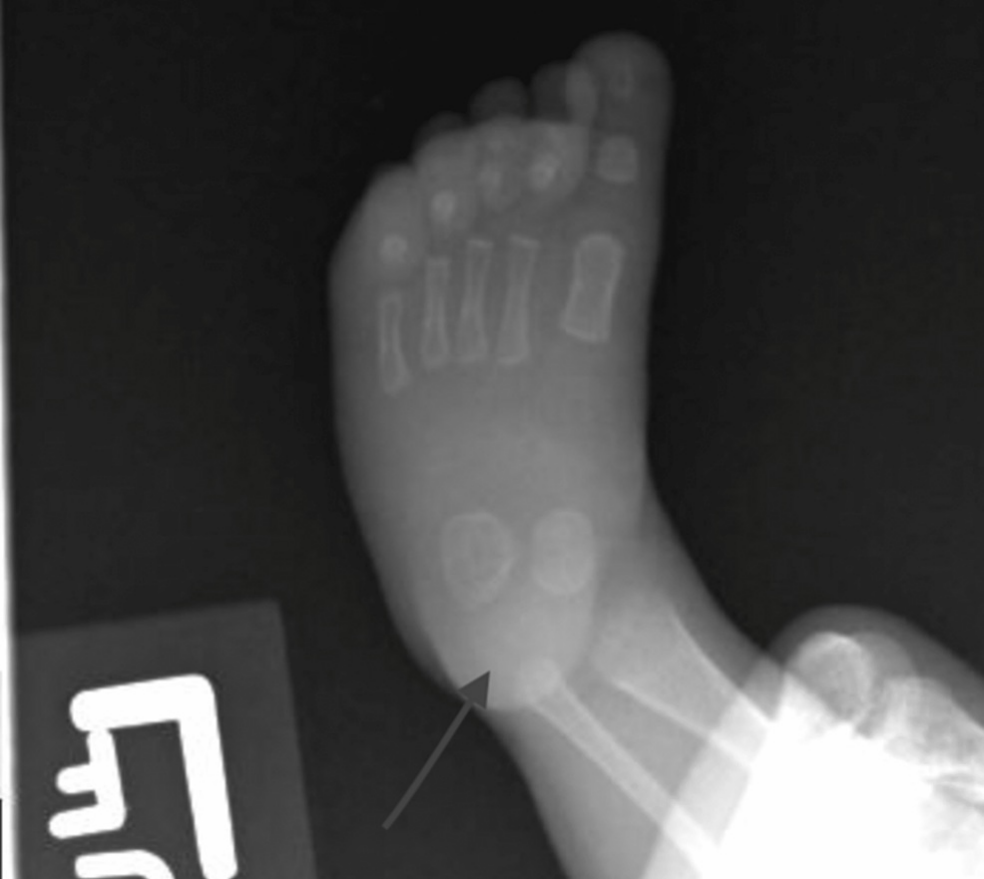
Arrow showing no signs of acute osteomyelitis of calcaneus

Doctors performed a repeat x-ray of the lower left extremity (Figure 2) after the patient had received one-month antibiotics in outpatient settings. This x-ray showed no signs of acute osteomyelitis in the calcaneus, and the outpatient immunology follow-up demonstrated IgG levels that continued to be low. The likely reason for the four-month-old patient’s low IgG was due to his prematurity and physiology. Maternal IgG is transferred in the third trimester, and as the patient did not reach the third trimester, he did not have a sufficient amount of IgG, though he had normal levels of IgA and IgM.

## Discussion

This report highlights the importance of recognizing the clinical manifestation of the disease, the risk factors, and the mode of transmission of infection to improve detection in newborns. Our patient was diagnosed with neonatal osteomyelitis of the calcaneus and multiple abscesses, with a significant antecedent of perinatal asphyxia, prematurity, and hypogammaglobulinemia. The route of spread occurred either locally from the surrounding infectious site or via direct inoculation of the organism during heel puncture blood sampling, leading to rapid spread through the cortex to the adjacent joint cavity.

Routine puncture wounds of the heel in neonates used to obtain blood samples normally enable healing without scarring, although the depth of penetration and the puncture site play an important role. A study by Blumenfeld et al. found that the punctures should be performed on the most lateral and medial aspects of the plantar surface of the heel. Avoiding the posterior curvature, the puncture should not be deeper than 2.4 mm [5]. The most common site of osteomyelitis is the metaphyses of the long bone, but osteomyelitis of the calcaneus is rare. The port of entry, in this case, was likely heel puncture, and very few cases have been reported in the literature [6-8]. Predisposing factors for calcaneus osteomyelitis include underlying disease, prematurity, low immunoglobulins, and heel puncture. The main components of the immune system are well established by 15 weeks of gestation. The source of protective plasma immunoglobulins in neonates is from transplacental maternal antibodies transferred initially. The transplacental antibody transfer of IgG occurs via active transport by the neonatal Fc receptor (FcRn) [9].

IgA and IgM do not bind to the FcRn; as a result, newborns are initially deficient in IgA and IgM [10]. The transplacental transfer of IgG occurs mostly in the third trimester of pregnancy [11-14]. At birth, the level of IgG is directly proportional to gestational age, leading full-term infants to have serum IgG levels comparable to maternal levels and premature infants to have lower IgG levels [13,15-17]. These lower levels of immunoglobulins predispose preterm neonates to recurrent and severe infections [18]. The diagnosis of neonatal osteomyelitis should be considered in the differential diagnosis when late-onset neonatal sepsis is suspected. The diagnosis of neonatal osteomyelitis in this infant was based on clinical presentation, laboratory findings, and radiological findings. The common changes seen on x-rays include periosteal thickening, lytic lesions, osteopenia, loss of trabecular architecture, and destructive bone changes appearing around 7-14 days of the disease. Antimicrobial treatment is frequently administered intravenously for two to three weeks and then switched to oral medication [18,19]. The need for longtime hospital admission is a common morbidity associated with neonatal osteomyelitis.

## Conclusions

The prematurity and immunity status of the infant, the mode and site of infection, and the isolated pathogen contribute to the rarity of the case reported. Osteomyelitis should be considered one of the differentials of late-onset sepsis in neonates. The local site of infection following a heel puncture relays the importance of using the correct method to perform blood draws.
